# Heterologous Production and Evaluation of the Biological Activity of Cystatin-B From the Red Piranha *Pygocentrus nattereri*

**DOI:** 10.3389/fgene.2022.812971

**Published:** 2022-06-03

**Authors:** Juan Antonio Ramirez Merlano, Daniela Volcan Almeida

**Affiliations:** ^1^ Aquaculture Institute, University of the Llanos, Meta, Colombia; ^2^ Laboratory of Molecular Biology, Institute of Biological Sciences, Federal University of Rio Grande, Rio Grande, Brazil

**Keywords:** aquatic diseases, fish, recombinant protein, transgene, stefins

## Abstract

Cystatin proteins are known to form a superfamily of cysteine protease inhibitors, which play a key role in protein degradation and are related to different physiological processes, such as development and immunity. Currently, numerous immunoregulatory proteins, such as cystatins, are being used in the control and prevention of diseases in aquaculture. Thus, the objective of this study was to produce recombinant cystatin (rCYST-B) from the red piranha *Pygocentrus nattereri* and to evaluate its effect on bacterial growth. The gene that encodes cystatin-B was isolated from the spleen of *P. nattereri* and cloned in an expression system. The protein was produced via a heterologous system involving the yeast *Pichia pastoris* X-33. The inhibitory activity of purified cystatin-B was evaluated on papain using different concentrations (0–80.0 μg/μL) of rCYST-B. The bacteriostatic action of the protein was evaluated using the *Kirby-Bauer* method on the growth of *Escherichia coli* and *Bacillus subtilis*. rCYST-B showed 100% inhibition at a concentration of 60 μg/μL. Moreover, the bacteriostatic activity of *E. coli* and *B. subtilis* showed inhibition of 40.36 and 49.08% compared to the negative control (phosphate buffer), respectively. These results suggest that recombinant CYST-B has biotechnological potential for use in aquaculture.

## Highlights

• Cystatin-B was successfully expressed in methylotrophic yeast, *Pichia pastoris* X-33.
• Functional analysis of purified rCystatin-B demonstrated papain inhibition activity.
• rCystatin-B efficiently inhibited the growth of *E. coli* and *B. subtilis.*
• Recombinant Cystatin-B exhibited potential for therapeutic use in fish.

## Introduction

Aquaculture is a productive sector with accelerated growth, an activity that has become fundamental for the achievement of the Sustainable Development Goals (SDGs) proposed by the United Nations (FAO 2017). According to the Food and Agriculture Organization of the United Nations (FAO) global fisheries production included approximately 179 million tons of fish, of which aquaculture represented production of 82.1 million tons, corresponding to 46% of the total production in 2018 (FAO SOFIA 2020). However, this growth was accompanied by the emergence or re-emergence of several infectious diseases (Pérez-Sánchez et al., 2018; FAO SOFIA 2020), such as acute hepatopancreatic necrosis (AHPND) (OIE 2013), enterocytozoon hepatopenaei (EHP) (Tang et al., 2017), emerging bacteria of the genus *Acinetobacter*, a potential pathogen in shrimp *Penaeus vannamei* (Huang et al., 2020) and some parasitic infections such as acanthocephalosis, reported for several groups of fish (Valladão et al., 2019), which have a detrimental influence on their health (Rosny et al., 2016). To overcome this problematic it is necessary to apply good management techniques and to develop new technologies. Biotechnology methods and genetic engineering enable the production of recombinant proteins, which can be used both in the prevention and treatment of diseases, and are considered promising for use in the aquaculture industry.

Cystatins are a large group of proteins that function as protease inhibitors involving cysteine residues in the proteolytic reaction (Berti and Storer 1995). Typically, they are small proteins that inactivate the protease substrate in a specific manner, forming reversible complexes (Turk and Bode 1991). Cysteines play essential roles in the physiology of all living organisms, from protozoa to mammals. In pathogenic microorganisms including bacteria, fungi, and parasites, cysteine protease can act as virulence factor, causing diseases in host organisms (Mottram et al., 2004; Rudenskaya and Pupov 2008). The cystatin superfamily is grouped into three families (Barrett 1986; Magister and Kos 2013), namely, family I (*Stefin*), family II (cystatins), and family III (kininogen). A fourth family has been reported of invertebrate origin, mainly of nematode parasites (Khaznadji et al., 2005; Li et al., 2010). Family I, composed of *Stefin* A and B, also known as cystatin A and B, are single-chain polypeptides of approximately 100 residues and molecular weight between 10 and 11 kDa without disulfide bonds or carbohydrate side chains. These are intracellular protease inhibitors present in the cytosol (Barrett 1986; Turk and Bode 1991; Abrahamson et al., 2003). Family 2 cystatins include cystatin C, D, E/M, F, G, S, SA, and SN with a molecular weight between 13–14 kDa and exhibit a signal peptide and two disulfide bridges (Cornwall and Hsia 2003). Family III (kininogens), are large glycoproteins (60–120 kDa) and complex in structure and are found in body fluids, especially in plasma (Barrett 1986; Prunk et al., 2016). All cystatins, regardless of family classification, contain several conserved regions, including an N-terminal glycine segment, a QXVXG sequence that constitutes part of the *β*-hairpin *loop* structure, and a proline-tryptophan-containing region that forms a second *hairpin loop* (Margis et al., 1998; Rzychon and Chmiel 2004). Cystatins play important defensive and regulatory roles during various cellular events. They modulate and stimulate TNF-α and IL-10 synthesis as a defense strategy in response to pathogen infection (Verdot et al., 1999).

In fish, cystatins exhibit protease inhibitory activity and appear to be involved in immune responses against infectious agents (Xiao et al., 2010; Premachandra et al., 2012; Ahn et al., 2013). To explore the functions of fish cystatin, some recombinant cystatins from Keta salmon (Yamashita and Konagaya 1991), trout (Li et al., 2000), and carp (Tzeng et al., 2001) were generated by heterologous expression in *Escherichia coli*. Although these recombinant proteins have already been developed for fish, the use of a species-specific protein or that obtained from a phylogenetically close species may decrease the side effects, besides potentiating the expected immunoregulatory effect.

Currently, genome sequencing allows the use of genetic information to produce specific proteins for application in aquaculture. However, few species of commercial interest have their genome completely sequenced. One such species is the red piranha *Pygocentrus nattereri*. The red piranha is a widely distributed carnivorous fish species that is observed in the rivers and lakes of South America (Behr and Signor 2008). In addition to being a popular aquarium fish, piranhas are often sold for human consumption in local markets in the Amazon basin (Duponchelle et al., 2007). Another important characteristic of this species is its similarity phylogenetic with other freshwater species such as tambaqui (*Colossoma macropomum* of the family Serrasalmidae). Associating the genetic knowledge of species of commercial interest to the development of biotechnologies allows the production of recombinant proteins of industrial interest, such as cystatin, for applications in aquaculture to maintain animal health. Thus, in this study, the main objective was the heterologous production and evaluation of the biological activity of the Cystatin-B derived from the red piranha *Pygocentrus nattereri.*

## Material and Methods

### Source of Animals and Sample Collection

Red piranha *Pygocentrus nattereri* juveniles, weighing approximately 200 g were captured at Janauacá Lake, municipality of Manaquiri - Amazonas, Brazil. The fish were euthanized (*n* = 13) with eugenol (200 mg/L), and the spleen tissue was harvested, preserved in RNAlater (Ambion, United States) and stored at −80°C.

### RNA Isolation and cDNA Synthesis

Total RNA extraction from the red piranha spleen samples was performed using the total RNA extraction protocol using Trizol Reagent ® (Invitrogen, United States), according to the manufacturer’s recommendations. The quality of the extracted RNA was evaluated by 1% (w/v) agarose gel electrophoresis and quantified using a spectrophotometer (BioDrop - Biodrop µLite). Total RNA was treated with the DNAse I (RQ1 RNase-Free DNase - Promega) following the protocol described by the manufacturer. Complementary DNA (cDNA) was generated using the High-Capacity cDNA Reverse Transcription Kit (Thermo Fisher Scientific, United States) from 1 µg of total RNA using oligo d (T) primer.

### Isolation of *Cystatin-*B Gene

Isolation of the genes encoding *cystatin*-B was performed by polymerase chain reaction (PCR). Specific primers were previously designed with the Primer-BLAST tool from GenBank (www.ncbi.nlm.nih.gov) based on the sequences XM_017717432 of *Pygocentrus nattereri* and used in this experiment. The primer sequences are listed as follows: Forward: 5′ CAG​CAG​GAG​AGC​AGA​AGT​TGA 3′ e Reverse 5′ TGT​TAG​TAC​GGT​TTG​TTA​AGG​GGA 3’. GoTaq® (Promega Corporation. Madison, United States) was used in the reaction, following the manufacturer’s recommendations. PCR cycles were set as follows: initial cycle at 94°C for 2 min, 35 cycles at 94°C for 30 s, 60°C for 30 s, and 72°C for 30 s, followed by a final extension at 72°C for 5 min.

After verifying the correct fragment size using electrophoresis in 1% agarose gel, the samples were purified using the PureLink™ PCR Purification Kit® (Invitrogen, Brazil), following the manufacturer’s protocol. The purified PCR products were sequenced using the Sanger method on the Applied Biosystems® Sanger Sequencing 3500 Genetic Analyzer. Automated sequencing was performed by the company ACTGene Molecular Analyses (UFRGS, Brazil). The sequencing result was first analyzed by the quality of the electropherograms obtained using the Chromas software and then aligned using the BLASTn tool (https://blast.ncbi.nlm.nih.gov).

### Bioinformatics Analysis

After confirmation by sequencing, protein analyzes were performed based on the predicted protein sequences using the Genebank database. Was used the cystatin-B isoform X1 (XP_017572921.1) protein. Multiple alignment analysis of the amino acid sequences of the CYSTATIN-B protein (named CYST-B herein) was performed using Geneious Prime Software (version 2020.1.2). The signal peptide deduced from the amino acid sequence was predicted by XtalPred (https://xtalpred.godziklab.org) and SignalP 5.0 Software (http://www.cbs.dtu.dk/services/SignalP). The secondary structure of the amino acid sequence of the protein was constructed using PredictProtein (https://predictprotein.org) and Phyre2 system (http://www.sbg.bio.ic.ac.uk/phyre2). The 3D structural model of the protein was established using the Phyre2 system. The phylogenetic tree was constructed based on the amino acid sequence of Cystatin-B, using MEGA X (Kumar et al., 2018) and the *Neighbour-joining* method with 1000 *bootstrap* replicates.

### Plasmid Construction

**FIGURE 1:**
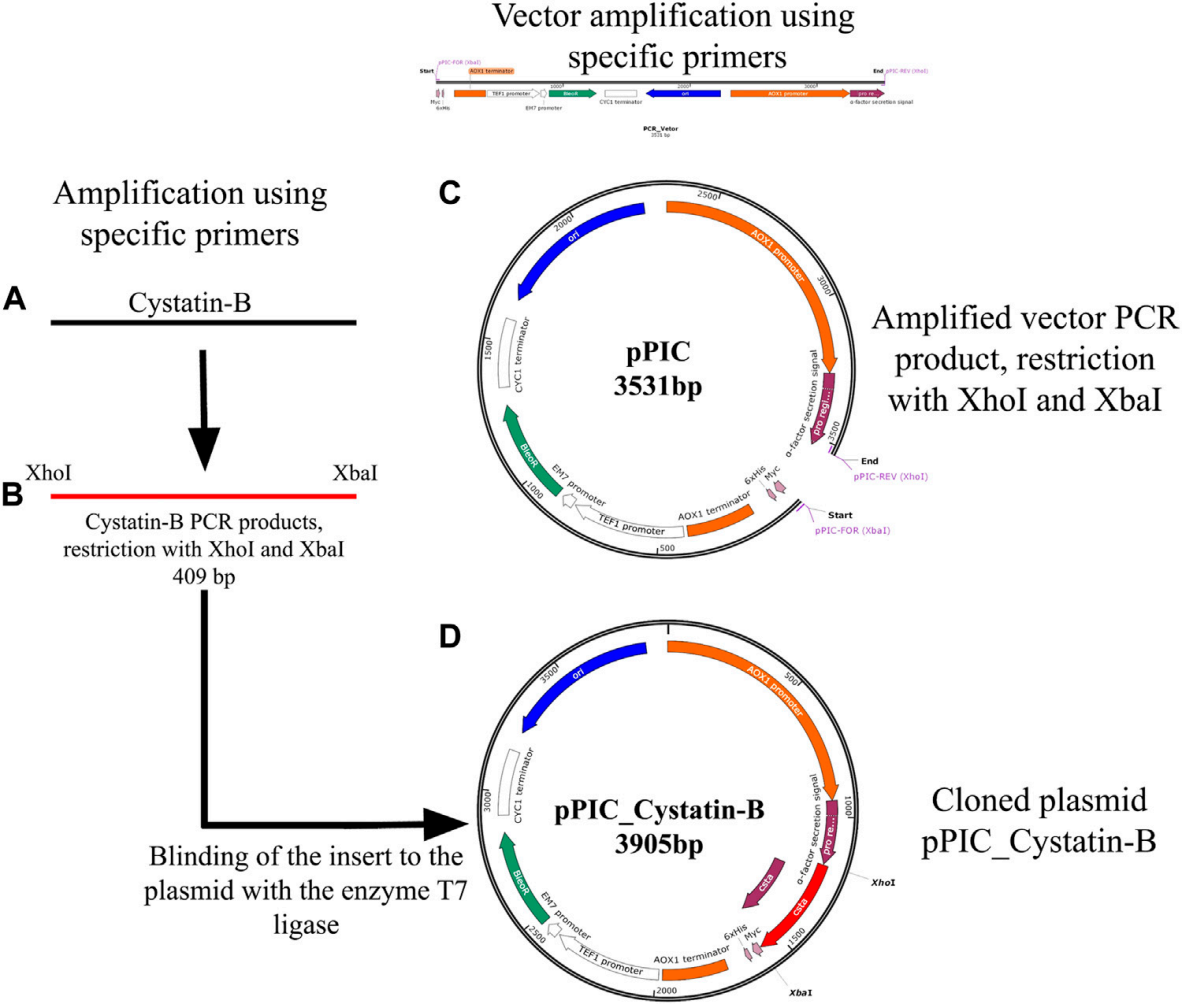
Schematic representation of the genetic construct containing *cystatin-B* **(A)** Amplification by PCR of the *cystatin-B* gene of juveniles of *Pygocentrus nattereri*, using specific primers, designed based on the sequence XM_017717432. **(B)** Amplified PCR products and cleaved with XhoI and XbaI enzymes. **(C)**. Amplified vector (pPIC) products by PCR, cleaved with XhoI and XbaI. **(D)**. Cloned plasmid pPIC_Cystatin-B (Binding of the insert to the plasmid with the enzyme T7 ligase).

Specific primers were re-designed by inserting restriction enzyme sites of XhoI and XbaI (cystBXhoI- For = 5′AAC​TCG​AGC​AGC​AGC​AGG​AGA​GCA​GAA​GTT​GA 3′ and cystBXbaI-Rev = 5′AAT​CTA​GAT​GTT​AGT​TAG​TAC​GGT​TTG​TTA​AGG​GGA 3′). The insertion of these sites allowed the cloning of fragments in the pPICZα expression vector (Figure 1). GoTaq® (Promega Corporation. Madison, United States) was used in the reaction, following the manufacturer’s recommendations. PCR cycles were set as follows: initial cycle at 94°C for 2 min, 35 cycles at 94°C for 30 s, 60°C for 30 s, and 72°C for 30 s, plus a final extension at 72°C for 5 min. Verification of the size of the PCR products was performed using agarose gel electrophoresis (1%). After confirmation, the PCR products were purified using the PureLink PCR Purification Kit (Thermo Fisher Scientific, United States) and cleaved using the enzymes XhoI and XbaI (Promega Corporation, United States), following the protocol described by the manufacturer (Figure 1B). The cleaved fragments were further purified (as described above) for binding to the pPICZα expression vector. The fragment vector was obtained by PCR using the pPICZα plasmid as the template (Figure 1C). The primers pPIC-Forward (XbaI) ATC​TAG​AAC​AAA​AAC​TCA​TCT​CAG​AGA​AGA​GG and pPIC-Rever (XhoI) AGC​TTC​AGC​CTC​TCT​TTT​CTC​GAG​AG were used. PCR conditions were set as follows: an initial cycle of 94°C for 2 min, 35 cycles of 94°C for 30 s, 60°C for 30 s, and 72°C for 4 min in addition to a final extension of 72°C for 10 min. The pPIC vector was also cleaved with the same restriction enzymes (XbaI and XhoI) to allow cohesive sequence formation and binding at complementary sites. The binding of the cleaved and purified insert to the vector was performed using the enzyme T7 ligase (Invitrogen, Brazil), following the manufacturer’s recommendations (Figure 1D).

### Transformation Into *Escherichia coli* With the Cloning Vector

The obtained genetic constructs were used for *Escherichia coli* TOP10 transformation. The calcium chloride method was used to generate chemically competent cells. Plasmid DNA was extracted using the plasmid DNA kit PureYield ™ Plasmid Miniprep System (Promega). After transformation, bacteria were grown overnight (37°C) in culture plates in LB (Luria-Bertani) solid medium and 50 μg/ml of the selection antibiotic Zeocin™ (Invitrogen, Brazil). *E. coli* colonies transformed with the cloned plasmid pPIC_cystatin -B were inoculated in LB liquid culture medium containing Zeocin (50 µg/ml^−1^) with shaking at 225 rpm and a temperature of 37°C. Plasmid DNA extraction was performed using the PureYield™ Plasmid Miniprep System plasmid DNA kit (Invitrogen, Brazil) according to the manufacturer’s recommendations. Plasmids extracted were cleaved with XhoI, XbaI, BamHI, and *Bg*LII and visualized using 1% agarose gel electrophoresis, as well as by PCR using different combinations of primers for specific regions both in the gene of interest and regions of the pPIC_cystatin B plasmid: 5′AOX- (3′ GAC​TGG​TTC​CAA​TTG​ACA​AGC 5′) and 3′AOX -(5′ GCA​AAT​GGC​ATT​CTG​ACA​TCC 3′).

### Cloning in the Yeast *Pichia pastoris*

Following verification, the plasmid was linearized using the restriction enzyme BamHI, the products were visualized by 1% agarose gel electrophoresis, quantified using a spectrophotometer (BioDrop, Isogen Life Science, Netherlands), and purified. Competent cells were prepared following the procedure in the Easy Select ™ Pichia Expression Kit manual (Thermo Fisher Scientific, United States). The transformation was performed in *P. pastoris* X33 yeast using the PichiaPink™ Expression System protocol (Thermo Fisher Scientific, United States) with some modifications (was used linearized plasmid DNA). A total of 80 μL of the cells with linearized plasmid DNA were mixed into electroporation cuvettes (0.2 cm) and incubated for 5 min on ice. They were subsequently electroporated (1500 V, 25 μF, 400 Ω). Immediately, 1 ml of ice-cold sorbitol was added to the cuvette, and the contents were transferred to a sterile 1.5 ml Eppendorf-type tube and incubated for 2 h at 30°C without shaking. A total of 100 μL of the transformed cells were plated onto Minimal Dextrose Medium (2% agar; 2% glucose; 4 × 10^-5^% biotin; yeast nitrogen base YNB 1.34% and 125 μg/ml Zeocin). The plates were incubated at 30°C for a maximum of 4 days until the development of colonies and selection of clones for further induction analyses.

### Expression and Purification of Recombinant Cystatin-B Protein

To increase the cell mass the selected clones and the negative control (without plasmid) of *P. pastoris* were inoculated in 200 ml of Buffered Glycerol Complex Medium (yeast extract 1%; peptone 2%; phosphate buffer pH 6.0 100 mM; YNB 1.34%; biotin 4 × 10^-5^%; glycerol 1%) and incubated at 30°C with shaking at 250 rpm until the culture showed an OD_600_ between 2–6, in approximately 18 h. Upon reaching the required OD_600_, the cultures were centrifuged at 3000 rpm for 10 min at 4°C and resuspended in 300 ml of Buffered Methanol Complex Medium (yeast extract 1%; peptone 2%; phosphate buffer pH 6.0 100 mM; YNB 1.34%; biotin 4 × 10^-5^%; methane 1%) in 1 L Erlenmeyer flask. The expression of alcohol oxidase enzyme promoter 1 (AOX1) was induced by adding absolute methanol every 24 h to the cell cultures, maintaining a final concentration of 1% (v/v) for 120 h at 30°C with shaking at 250 rpm. Aliquots containing 50 ml of the cultures were collected, centrifuged at 3000 rpm for 10 min at 4°C, and preserved at −80°C for further analysis. The recombinant protein was purified using Ni Sepharose high-Performance nickel-based resin (GE Healthcare, United States) with polyhistidine tail (6xHis). The purified recombinant rCYST-B protein was analyzed via denaturing 15% polyacrylamide gel electrophoresis (SDS-PAGE). Subsequently, the gels were stained with Coomassie blue (0.25%) (Figure 3A), and silver nitrate (20%) (Figure 3B). The concentration of the purified recombinant protein was evaluated according to the method described by Lowry et al. (1951).

### Protease Inhibition Assay

The inhibitory effect of the recombinant rCYST-B protein was analyzed using papain as a cysteine protease, performed as described in Xiao et al. (2010) modified for (Yu et al., 2019). The assay was performed using different amounts of rCYST-B proteins (0, 0.1; 0.5; 1.0; 10.0; 15.0; 20.0; 30.0; 60.0 and 80.0 μg), which were incubated with 10 μL of papain (0.1 μg/μL) (Sigma-Aldrich, United States)at 28°C for 30 min. The reaction was initiated by adding 200 μL of Azocasein (0.2% W/V) (Sigma-Aldrich, United States), followed by incubation at 37°C for 2 h. The reaction was inactivated with 200 μL of 10% trichloroacetic acid (TCA). The contents were chilled on ice for 15 min. The precipitate was separated by centrifugation at 15,000 rpm for 5 min. The absorbance was evaluated by spectrophotometry at 450 nm. As a negative control, αGHT protein was used in place of rCYST-B. The relative activity of rCYST-B was determined by: 100x (1–(_A440_ of rCYST-B)/(_A440_ control).

### Bacteriostatic Action Test

The assay for the identification of bacteriostatic action was performed using the *Kirby-Bauer* method (Bauer et al., 1966). Two types of bacteria (*Escherichia coli* or *Bacillus subtilis*) were plated on the surface of Petri dishes with LB medium and incubated at 37°C for 12–16 h. Disks were added with rCYST-B (60 and 80 μg/μL), Spectinomycin 50 ug/uL (positive control), and Potassium Phosphate Buffer 50 mM (negative control). The bacteriostatic action was evaluated qualitatively and quantitatively. The qualitative analysis was related to the presence of inhibition halos around the disk, and the quantitative analysis evaluated the halo diameter (mm), attributed to the bacteriostatic action of the protein. For halo diameter analysis, the plates were photographed (*n* = 4) and analyzed using ImageJ software.

### Statistical Analysis

All experiments were performed in at least triplicates. All values are shown as mean ± standard deviation of the mean. Factorial type ANOVA was applied between the different treatments used to determine the bacteriostatic effect of rCYST-B; significant differences were determined with a *p* < 0.05. The analyses were performed using GraphPad Prism 8.0 software (GraphPad Software).

### Ethical and Legal Aspects

All procedures adopted in this study were performed according to the protocols previously approved by the Ethics Committee on Animal Use (CEUA) of Universidade Nilton Lins with approval protocol 015/2017.

## Results

### Characterization of CYST-B Structure

**FIGURE 2:**
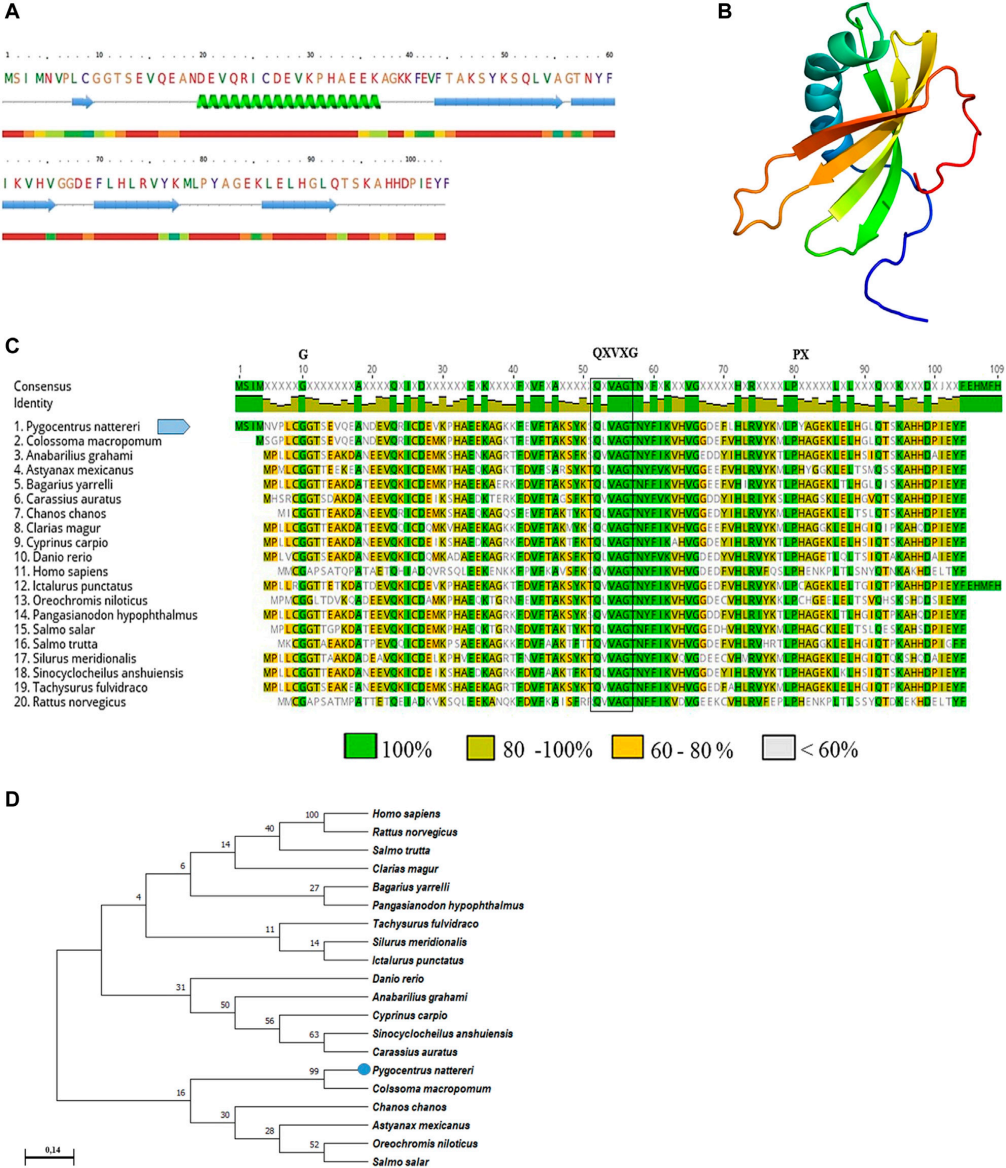
Bioinformatic analysis of *Pygocentrus nattereri* cystatin-B **(A)**. Secondary structure of cystatin-B of *P. nattereri* generated by PredictProtein and Phyre2 system. fx1 Alpha helix, fx2Beta Strand. **(B)**. The 3D tertiary structure of Cystatin-B generated by the Phyre2 system. Model dimensions (Å): X:44,068 Y:34.294 Z:30.445. **(C)**. Multiple alignments of the cystatin-B amino acid sequence of *Pygocentrus nattereri* and other sequences. *Colossoma macropomum,* XP_036414104.1; *Anabarilius grahami,* ROK23366.1*; Astyanax mexicanus,* XP_007249105.1; *Bagarius yarrelli,* TSK98422.1; *Carassius auratus*, XP_026093937.1; *Chanos*, XP_030625480.1; *Clarias magur*, KAF5903616.1; *Cyprinus carpio*, XP_018952861.1; *Danio rerio*, NP_001096599.1; *Homo sapiens*, NP_000091.1; *Ictalurus punctatus*, XP_017308724.1; *Oreochromis niloticus*, XP_003443657.1; *Pangasianodon hypopthalmus*, XP_026793986.1; *Salmo salar*, XP_014062104.1; *Salmo trutta*, XP_029625791.1; *Silurus meridionalis*, KAF7691695.1; *Sinocyclocheilus anshuiensis*, XP_016296734.1; *Tachysurus fulvidraco*, XP_027030842.1; *Rattus norvegicus*, NP_036970.1. Characteristic and conserved residues of glycine (**G**) and proline (P) are indicated by the letter “G” and “P”, respectively. The characteristic and preserved residues of the QXVXG motif are marked with a box. **(D)**. Phylogenetic tree of cystatin-B and sequences of their counterparts constructed using the neighbor-joining method and 1000 bootstrap replicates. The numbers presented in the branches represent the bootstrap value (%).

The PCR amplified gene products presented a fragment of 409 base pairs (bp) with 100% similarity to the partial sequence available in GeneBank (XM_017717432.2). The sequence of CYST-B showed 104 amino acid residues, with an estimated molecular weight of 11.755 kDa, a theoretical isoelectric point of 6.04, stability index of 33.98, without the presence of a signal peptide. The secondary structure analysis of CYST-B (Figure 2A), showed a structure composed of: *loop* 49.0%, helix 17.3%, and strand 33.7%. The 3D structure of CYST-B was predicted by Phyre2 (Figure 2B). The tertiary structure of CYST-B was colored using the rainbow command, blue at the N-terminal and red at the C-terminal. The rainbow command colors residues; 99 residues were modeled up to 100.0% confidence and a reliable model was obtained with the following characteristics: (Å): X: 44.068 Y:34.294 Z:30.445. Multiple homologous alignments of the amino acid sequences of CYST-B from *P. nattereri* (Figure 2C) revealed conserved regions typical of the cystatin superfamily, including two conserved N-terminal glycine residues (G10G11) as well as the glutamine-valine-glycine motif (QXVXG), crucial for the biological activity of the molecule. The motif represented by glutamine-leucine-valine-alanine-glycine (Q52LVAG56) exhibited homology with species such as *Colossoma macropomum* (GeneBank accession code XP_036414104.1) with 100% identity. In addition, a typical proline-tryptophan (PW) motif was identified at the C-terminus, where the tryptophan residue was replaced by a tyrosine residue (P80Y81). Based on the total amino acid sequence length of CYST-B, phylogenetic tree analysis was performed using MEGA X (Figure 2D). The result indicated a sequence similarity to that of tambaqui *Colossoma macropomum*, a phylogenetically close species.

### Expression and Purification of the Recombinant rCYST-B Protein

**FIGURE 3:**
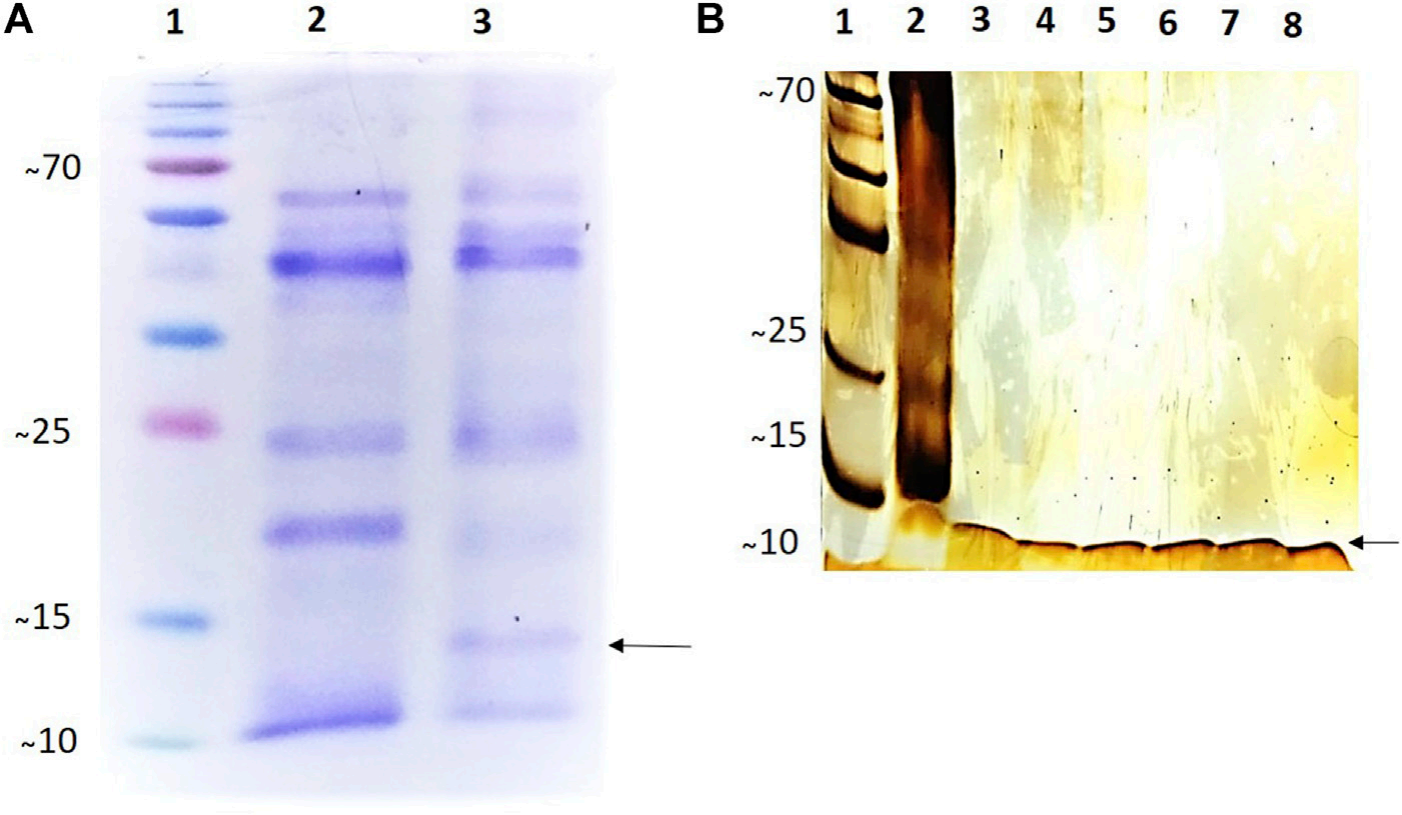
Analysis of the expression and purification of rCystatin-B with SDS-PAGE (120 h of induction). **(A)**. Gel stained with 0.25% Coomassie blue; (Lane 1). PMM - Molecular Mass Standard - kDa (Invitrogen). (Lane 2). Lyophilized fraction (crude extract) of *Pichia pastoris* cell culture without cloned plasmid (negative control) subjected to the induction and expression process; (Lane 3). Transformed and lyophilized fraction of the culture of *Pichia pastoris* cells subjected to the induction and expression process (inclusion of cloned plasmid: pPIC_Cystatin-B). **(B)**. Gel stained with Silver Nitrate (20%). (Lane 1). PMM - Molecular Mass Standard - kDa (Invitrogen). (Lane 2). Transformed and lyophilized fraction of the culture of *Pichia pastoris* cells subjected to the induction and expression process (inclusion of cloned plasmid: pPIC_Cystatin-B). (Lanes 3–8). Purified rCystatin-B protein (indicated by the arrow). The recombinant protein was purified using Ni Sepharose High-Performance Nickel based resin.

SDS-PAGE showed that purified rCYST-B produced a band (Figure 3B, Lanes 3–8) with the expected size of approximately 11.8 kDa. These results confirm the expression of the recombinant rCYST-B protein in *P. pastoris* X33 cells (Figure 3A), demonstrating a suitable platform for the production of heterologous cystatin proteins.

### Inhibitory Activity

**FIGURE 4:**
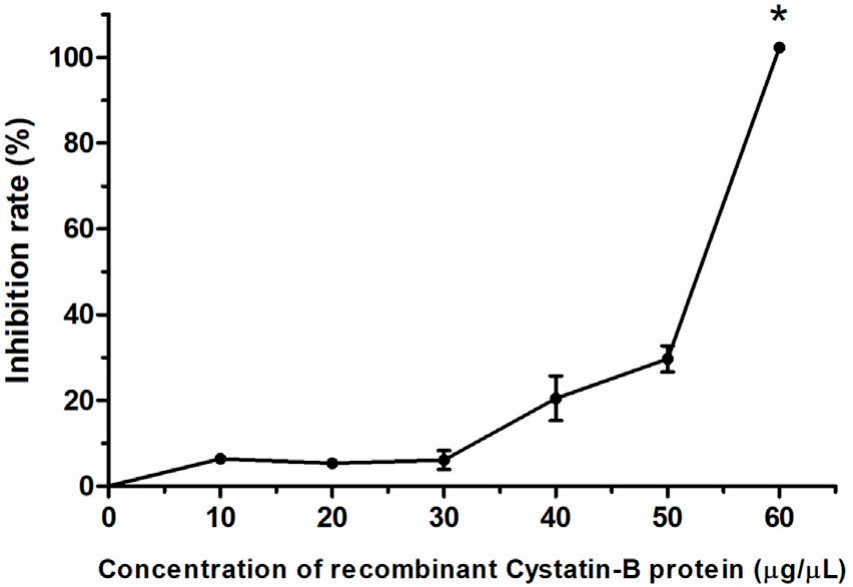
Inhibition pattern of rCystatin-B against papain activity. The curve represents the change in residual papain activity along with the addition of rCystatin-B. αGTH (negative control), did not show any inhibitory effect (data not shown). Values are shown as mean ± standard deviation from the mean (*n* = 3). Asterisc (*) indicate statistical difference (*p* = 0.003).

The *in vitro* inhibitory activity of the recombinant rCYST-B protein on papain (a cysteine protease), revealed a concentration-dependent inhibition response of the inhibitor, with the increase starting at a concentration of 30 μg/μL and reaching 100% inhibition at the concentration of 60 μg/μL (Figure 4).

### Analysis of Growth Inhibition and Bacteriostatic Action

**FIGURE 5:**
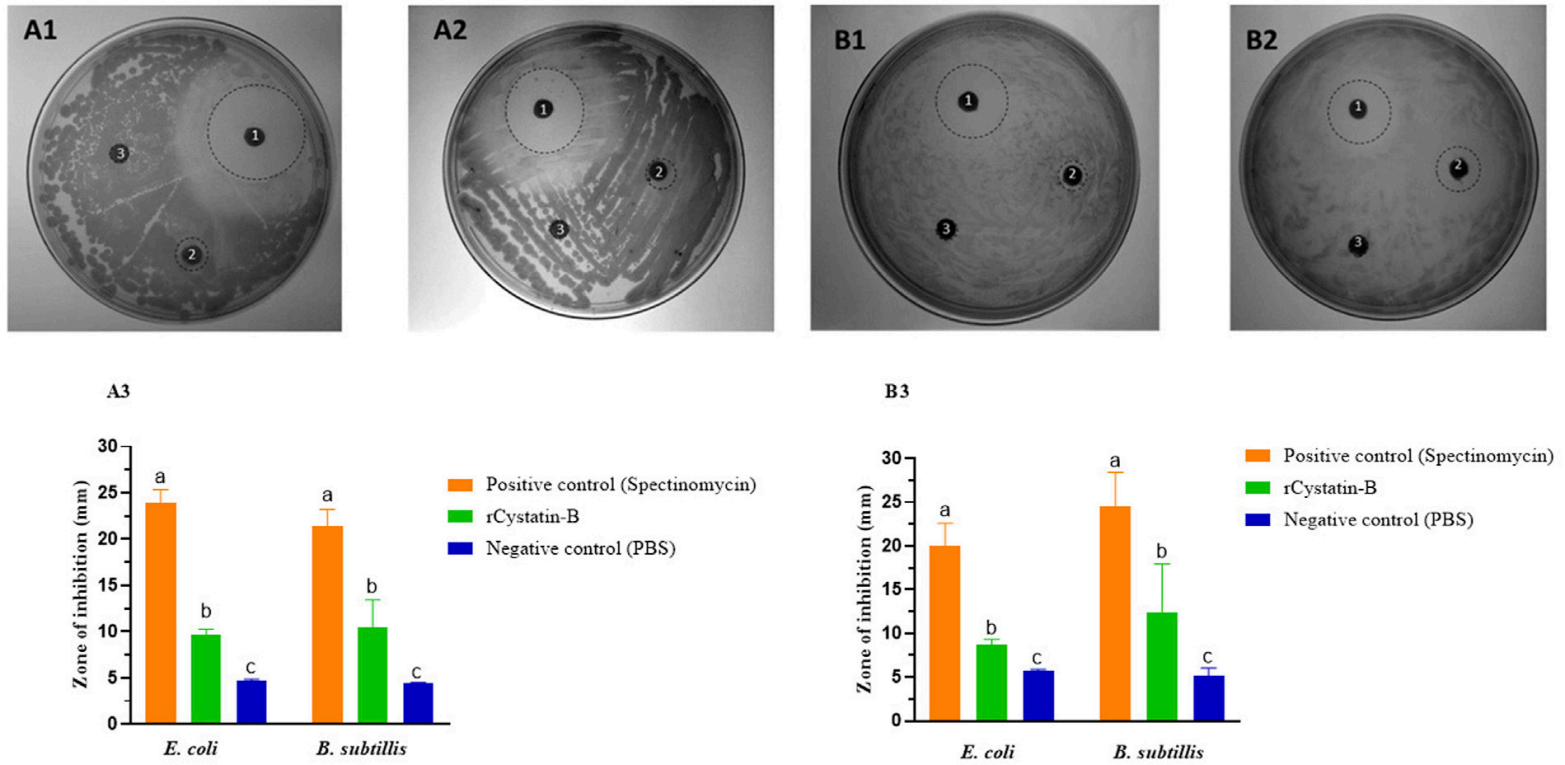
Bacteriostatic action of rCystatin-B. (**A**). 60 μg/μL. (A1). *E. coli.* (A2). *B. subtilis*. (A3). Zone of inhibition (diameter mm). (**B**). 80 µg/µL. (B1). *E. coli*. (B2)*. B. subtilis*. (B3). Inhibition diameter (mm). The dashed line circles correspond to the observed zone of inhibition. 1). Spectinomycin (positive control). 2) recombinant rCystatin-B protein. 3). PBS (negative control). Values are shown as mean ± standard deviation from the mean (*n* = 3). Different letters show statistical differences (*p* < 0.05).

The bacteriostatic activity of the recombinant rCYST-B protein on *E. coli* (Gram-negative bacteria) and *B. subtilis* (Gram-positive bacteria) demonstrated the presence of inhibition halos (zone of inhibition) at concentrations of 60 and 80 μg/μL of rCYST-B (Figure 5A). The diameter of the inhibition halo at the concentration of 60 μg/μL was 9.66 ± 0.61 mm for *E. Coli* and 10.50 ± 2.94 mm (*p* > 0.05) for *B. Subtilis* (Figure 5B). At the concentration of 80 μg/μL, the halo diameter was 8.64 ± 0.70 mm and 12.41 ± 5.53 mm for *E. coli* and *B. subtilis*, respectively (Figure 5C). These values represent a bacteriostatic action in comparison to the negative control (PBS), which did not show a halo of inhibition for any of the replicates. The positive control (spectinomycin antibiotic) showed a halo with a diameter of 23.93 ± 1.42 mm and 21.39 ± 1.83 mm at a concentration of 60 μg/μL for *E. coli* and *B. subtilis*, respectively. At the concentration of 80 μg/μL, the halo of inhibition in the positive control was 20.04 ± 2.55 mm and 24.53 ± 3.91 mm for *E. coli* and *B. subtilis*, respectively.

## Discussion

Developing new strategies for disease management and control is one of the current challenges in aquaculture. Biotechnology is considered an important tool for the treatment of emerging diseases associated with aquaculture production. In this study, the objective was to produce a protein with possible immunoregulatory potential for fish. For this, a 409 bp fragment related to the *cystatin* B gene was isolated from the spleen of the fish *Pygocentrus nattereri*. This is the first report of the isolation of this gene for this species.

Typically, cystatins in fish are composed of approximately 100 amino acids, varying somewhat according to the species and isoform. For example, in species such as *Scophthalmus maximus* (Xiao et al., 2010), the gene size of *cystatin* B was 300 bp length. Ahn et al. (2013) reported the stefin B gene with a size of 297 bp for *Paralichthys olivaceus*. Cystatin homologs or *stefin* A have been isolated from species such as *Ctenopharyngodon idella* with a nucleotide sequence length of 294 bp (Li et al., 2021). Another important feature of cystatins involves the three conserved domains that form the inhibitory sites of interaction with proteases: an N-terminal glycine, a glutamine-X-valine-X-glycine motif, and a C-terminal proline-tryptophan amino acid pair (Bode et al., 1988; Stubbs et al., 1990). In the present study, multiple alignments identified three conserved regions typical of the cystatin superfamily. In rCYST-B, the N-terminal domain containing the conserved glycine residue (G10G11) and the presence of similarity to its homologs from other species were found. The 2 G residues are known to constitute a wedge-shaped border, involved in the inhibition of protease activity, complementary to the active site of papain-like cysteine proteases (Bode et al., 1988; Abrahamson 1993). In addition, it is the domain involved in the interaction between the protease and the inhibitor, which allows it to interact directly with the S3, S2, and S1 substrate-binding regions of the protease (Hall et al., 1995; Bjork et al., 1995). The QXVXG motif, highly conserved in cystatins, was observed in rCYST-B from *P. Nattereri*. This motif binds to the active site of cysteine proteases, thereby interfering with their interaction with the substrate and inhibiting their activity (Bode et al., 1988; Turk and Bode 1991). The variation in the presence of amino acids proline - tyrosine in the C-terminal region of Cystatin B theoretically does not affect the inhibitory activity, both amino acids have hydrophilic characteristics and very similar isoelectric points. The inhibitory response was observed in the recombinant rCYST-B produced in the present study. Different types of C-terminal variations have been observed in human *Stefin* B, with replacements by the histidine (H) residue (Bjork et al., 1995) as well as in *Acipenser sinensis* (Bai et al., 2006) with the replacement by the Leucine (L) residue. Analysis of the phylogenetic tree performed in this study (Figure 2D) confirms the evolutionary relationship and proximity of Cystatin-B to its cystatin homologs as well as the functions among type 1 cystatin (*Stefins*) (Pemberton 2006).

The estimated molecular weight of rCYST-B was 11.8 kDa, similar results are observed for recombinant cystatin B protein obtained from *Ctenopharyngodon idella* with 11.48 KDa (Li et al., 2021), *Pseudosciaena crocea* with 11.4 KDa (Li et al., 2009), *Scophthalmus maximus* with 11.1 kDa (Xiao et al., 2010), *Paralichthys olivaceus* with 12.7 KDa (Ahn et al., 2013), reported values for *Stefins* family cystatins (Ochieng and Chaudhuri 2010).

After bioinformatics analyses, we evaluated the protease inhibition activity of the purified recombinant CYST-B protein. For this, we chose papain as cysteine protease and azocasein as substrate. The rCYST-B from *P. Nattereri* exhibited concentration-dependent inhibition of papain activity. These results confirmed that the purified recombinant protein was able to maintain its biological functions. The concentration at which 100% inhibition was observed was 60 μg/μL. However, a previous study reported a concentration of 0.5 μg/μL for *Stefins* B homologs from *Oplegnathus fasciatus* (Premachandra et al., 2012). Recently, Li et al. (2021), reported a dose-dependent inhibition pattern for the proteolytic activity of papain, cathepsin B, and cathepsin L, showing a decrease in the activity with the increasing amount of *Stefin* A isolated from *Ctenopharyngodon idella*. In the case of species such as *Crassostrea gigas*, the concentration of 50 μg/μL of cystatin A showed an inhibition rate of around 80% (Mao et al., 2018). In contrast, Premachandra et al. (2012), produced and purified a recombinant cystatin B from rock bream *Oplegnathus fasciatus* and demonstrated 82% inhibition of papain activity at a concentration of 0.5 μg/μL. Even though there is a wide variation in the concentration of cystatin showing 100% inhibition of protease activity, all results suggest that these recombinant proteins may show regulation of exogenous proteases from microorganisms and parasites that infect the host (Scott et al., 2007). These differences in the potential or response of the inhibitory activity of cystatin are supported by the wide spectrum of its structural variation and distribution in different types of cells and tissues (Lefebvre et al., 2008; Xiao et al., 2010); structure characterized by highly conserved regions that form a discrete wedge-shaped structure that blocks the active site of cysteine proteases (Gregory and Maizels, 2008). On the other hand, G11 introduces some flexibility in the N-terminal region of inhibitors, allowing the N-terminal motif to adopt an optimal conformation for enzyme-inhibitor interaction (Hall et al., 1993). The greater inhibitory potential exerted on cysteine proteases has allowed the use of the specificity of Cysteine protease inhibitory activity substrates, as well as different evaluation protocols under *in vitro* conditions (temperature and enzymatic reaction times) (Bai et al., 2006; Xiao et al., 2010; Wickramasinghe et al., 2020).

In our study, it was evidenced that the protein heterologously produced by *P. pastoris* was able to inhibit the growth of *E. coli* and *B. subtilis.* Inhibition of *E. coli* and *B. subtilis* bacterial growth were also observed with *Paralichthys olivaceus* Cystatin-C (Yu et al., 2019) as well as on *Scophthalmus maximus* Cystatin-B (Xiao et al., 2010). Previous results confirm the antiviral and antibacterial role of cystatins (Wesierska et al., 2005). In general, proteins with antimicrobial capacity use their own cationic charge to attack and enter in a faster and more effective way through the bacteria membrane, increasing membrane permeability and flow of intracellular contents and subsequently, cell lysis (Izadpanah and Gallo 2005). We believe that this mechanism is used by rCYST-B, however, further research is needed to prove this affirmation. It should be noted that Cystatin-B is involved in immune responses to bacterial and fungal infections and anti-apoptotic processes in the brain (Takahashi et al., 1994; Di Giaimo et al., 2002). They are also expressed at high levels in follicular dendritic cells, which are involved in the immune and inflammatory reaction, present in the germinal centers of secondary lymphoid organs (Rinne et al., 1986). Furthermore, they can prevent apoptosis of B cells containing high-affinity receptors for the antigen present on the surface of follicular dendritic cells (van Eijk and Groot 1999).

Based on the confirmation of the antibacterial potential of rCYST-B, we believe that this protein can be used as an immunoregulatory protein for fish, and also as a food supplement in the fish feed. This hypothesis is based on the fact that recombinant fish cystatins have already been produced and showed potential in preventing fish diseases (Bjorklund et al., 1997). Recombinant cystatins obtained from Keta salmon (Yamashita and Konagaya 1991), trout (Li et al., 2000), and carp (Tzeng et al., 2001) were generated by heterologous expression in *Escherichia coli*. Even though these recombinant proteins have already been developed for fish, the use of a species-specific protein or that obtained from a phylogenetically close species may decrease the side effects besides potentiating the expected immunoregulatory effect.

The results of this study constitute the first reports on the production of a recombinant rCYST-B protein for a species native to South America. In conclusion, the systematic study of cloning, expression, and characterization of the biological activity of the recombinant cystatin B protein from *Pygocentrus nattereri* suggests its role as a possible immunoregulatory protein in biological defense against invaders and cellular protection against proteolysis mediated by cysteine proteases.

## Data Availability

The original contributions presented in the study are included in the article/Supplementary Materials, further inquiries can be directed to the corresponding author.
