# Supplementary figures and images for: Heterologous Production and Evaluation of the Biological Activity of Cystatin-B From the Red Piranha Pygocentrus nattereri
Source: Front Genet. 2022 Jun 3;13:812971. doi: 10.3389/fgene.2022.812971 (PMC9203827; doi:10.3389/fgene.2022.812971)

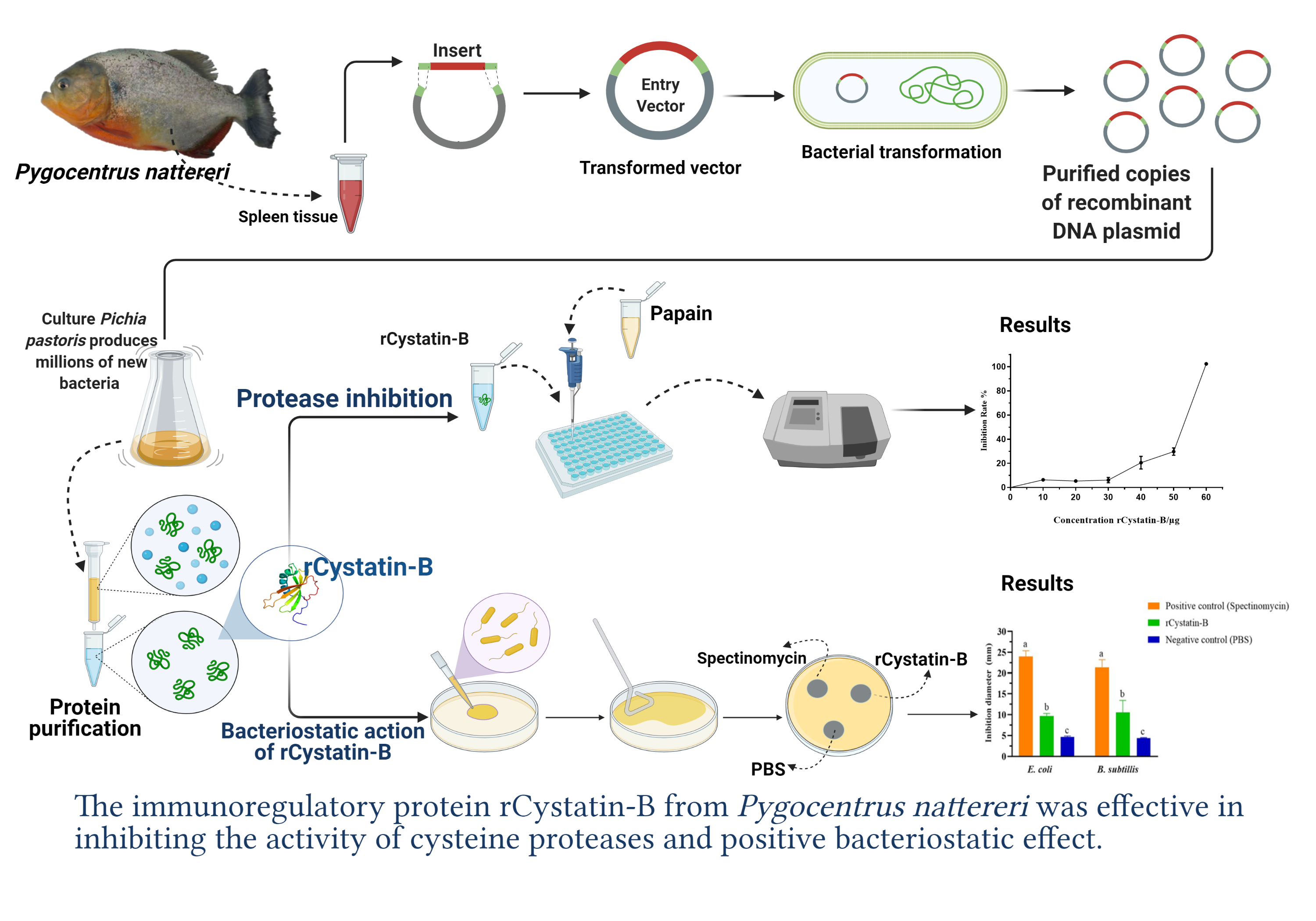

Supplement: Supplementary file 1 [file Image1.PNG]
